# Biomechanical comparative analysis of multiple small diameter fan-shaped and parallel core decompression for early osteonecrosis of the femoral head

**DOI:** 10.3389/fbioe.2026.1773122

**Published:** 2026-04-29

**Authors:** Daizhu Yuan, Jianya Qiu, Zhonghui Yao, Cheng Wu, Jiayi Luo, Chuan Ye

**Affiliations:** 1 Department of Orthopaedics and Sports Medicine, The Affiliated Hospital of Guizhou Medical University, Guiyang, China; 2 Department of Orthopedics, Guizhou Hospital, The First Affiliated Hospital of Sun Yat-Sen University, Guiyang, China; 3 Center for Tissue Engineering and Stem Cells, Guizhou Medical University, Guiyang, China

**Keywords:** biomechanical stability, core decompression, finite element analysis, osteonecrosis of the femoral head, proximal femur

## Abstract

**Background:**

Multiple small diameter fan-shaped and parallel core decompression are the surgical procedure used to treat the early osteonecrosis of the femoral head. However, It is not known which method is more appropriate from biomechanical perspective. Therefore, we aimed to analyze the mechanical stability of different distribution parameters of the two drilling methods using finite element analysis.

**Methods:**

Ten finite element models were established, including different drilling number (three-hole, four-hole, five-hole, seven-hole and nine-hole) based on fan-shaped and parallel core decompression. The stress and strain of the proximal femur was calculated to evaluate the biomechanical stability.

**Results:**

Results showed that maximum equivalent stress in the entire structure, greater trochanter’s lateral wall, and bone tunnels increased with more drill holes in both groups, following the order: three-hole < four-hole < five-hole < seven-hole < nine-hole. At the same hole count, fan-shaped core decompression had higher maximum equivalent stress in the greater trochanter’s lateral wall, cortical bone tunnel, cancellous bone tunnel, and bone tunnel in the osteonecrosis area compared to parallel core decompression, with notable percentage increases in the cancellous bone tunnel (21.18%–585.71%). Additionally, fan-shaped drilling exhibited higher average stress and local strain, especially in the cancellous bone tunnel, whereas parallel drilling maintained lower stress and strain levels across most regions.

**Conclusion:**

From a biomechanical perspective, multiple small-diameter parallel core decompression is superior to fan-shaped decompression, offering better stability and reduced stress concentration in the proximal femur for early femoral head osteonecrosis.

## 1 Introduction

Osteonecrosis of the femoral head (ONFH) is a common refractory disease in clinical practice (George and Lane, 2022). Hip trauma, prolonged use of corticosteroids, and chronic excessive alcohol intake are common pathogenic factors of ONFH (Ko et al., 2023; Migliorini et al., 2023). If untreated properly, the advanced stage may be characterized by progressive femoral head collapse, leading to deformity and hip joint dysfunction, thereby severely affecting patients’ quality of life. Therefore, early intervention aimed at preserving hip function before femoral head collapse is of critical importance (Ng et al., 2023).

Core decompression is a commonly used method for treating early ONFH, which can be divided into single channel large-diameter core decompression and multiple small diameter core decompression (Yue et al., 2022; Mont et al., 2004). The primary advantage of single channel large-diameter core decompression lies in its operational simplicity. However, the effectiveness of decompression is directly constrained by the drill diameter, making it difficult to cover extensive areas (Hua et al., 2019). Furthermore, increasing the drill diameter to expand the decompression zone may excavate critical structural bone tissues, creating voids that disrupt local stress distribution, potentially leading to stress concentration (Yuan et al., 2022a). This disruption significantly weakens the mechanical support provided by the subchondral bone. When the cumulative bone damage exceeds the tissue’s repair capacity, it ultimately elevates the risk of femoral head collapse (Kurup et al., 2025). To mitigate the impact of single channel large-diameter core decompression on the biomechanical strength of the proximal femur, some studies have introduced multiple small diameter core decompression (Mont et al., 2004). This approach creates a network of tunnels within the necrotic area, preserving inter-tunnel bony bridges to maintain structural integrity (Brown et al., 2016). The multi-hole configuration optimizes biomechanical performance through three key mechanisms: (1) Stress redistribution: By dispersing concentrated loads across surrounding bone tissues, this technique significantly reduces peak stress compared to single-channel methods; (2) Structural continuity: The preserved bony bridges maintain the mechanical conduction pathway between cortical and trabecular bone, ensuring sustained vertical support stiffness in the subchondral region; (3) Enhanced decompression efficiency: While individual holes have a smaller decompression radius, their collective arrangement covers a larger necrotic area, minimizing stress concentration around bone tunnels. Although the multiple small diameter core decompression technique can reduce the impact on the biomechanics of the proximal femur compared with the large-diameter single-channel core decompression technique, its narrow drilling bone tunnel limits the function of combining other treatment methods, such as platelet-rich plasma (Xu et al., 2024), stem cells (Wang et al., 2019), and bone grafting (Mei et al., 2023).

At present, the multiple small diameter core decompression include the fan-shaped and parallel core decompression (Zhao et al., 2024; Li et al., 2017). The multiple small diameter parallel core decompression involve arranging multiple bone tunnels in a parallel manner, creating a series of regular circular bone holes on the greater trochanter’s lateral wall. The multiple small diameter fan-shaped core decompression is distributed in a fan-shape (all core decompression converged at a single hole on the greater trochanter’s lateral wall) and constructed different numbers of small-diameter core decompression (Zhao et al., 2024). However, It is not known which method is more appropriate from a biomechanical perspective. The drilling position of core decompression is the lateral wall of the femoral trochanter, and multiple porous channels are drilled through Kirschner wires. The lateral wall of the femur is cortical bone, which is denser in texture compared to cancellous bone, with strong compressive and torsional resistance, and plays a crucial role in the mechanical support of the entire body (Fleps et al., 2020). Core decompression damages the cortical bone of the lateral wall, which may cause disruption of the original mechanical structure and decrease in mechanical strength (Yuan et al., 2022b). Moreover, studies have shown that the integrity of the lateral wall of the trochanter is of great significance for maintaining the biomechanical stability of the proximal femur (Li et al., 2023). The characteristic of parallel drilling is to form regular circular channels in the lateral wall of the trochanter and regular parallel channels in the femoral head and neck. The fan-shaped drilling not only forms irregular bone tunnels in the femoral head and neck, but also inevitably leads to irregular morphology at the entrance of the bone tunnel due to sharing a common bone channel on the greater trochanter’s lateral wall. According to the stress-strain theory, irregular morphology is more prone to causing local stress shielding and stress concentration during stress transmission (Welch-Phillips et al., 2020). Whether this scenario applies to fan-shaped drilling requires further investigation.

Therefore, we conducted this study to analyze the mechanical stability of different distribution parameters of the multiple small-diameter fan-shaped and parallel core decompression using finite element analysis methods and provide some theoretical references for clinical application.

## 2 Materials and methods

### 2.1 Construction of three-dimensional (3D) femur model and early ONFH

**FIGURE 1:**
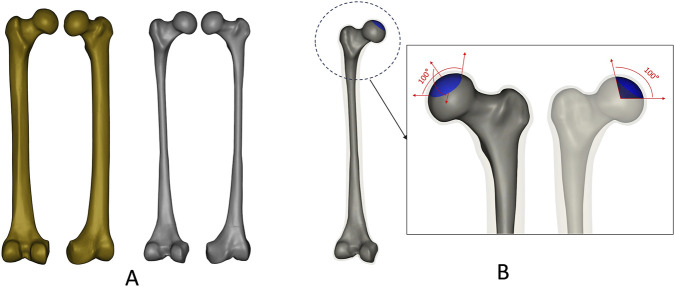
Construction of 3D models. **(A)** Femoral cortical and cancellous bone; **(B)** Necrotic area of early ONFH.

A 3D model of the Sawbones® left 4th-generation composite femur (Model 3406; Sawbones, Vashon, WA) was used for the femoral geometric model, including cortical and cancellous bones (Figure 1A). Early ONFH was constructed using SolidWorks 2022 (Dassault, France), with the necrosis area defined by 100° angles in the midcoronal and midsagittal femoral planes, corresponding to early ONFH (Koo and Kim, 1995) (Figure 1B).

### 2.2 Construction of different distribution models based on fan-shaped and parallel core decompression

**FIGURE 2:**
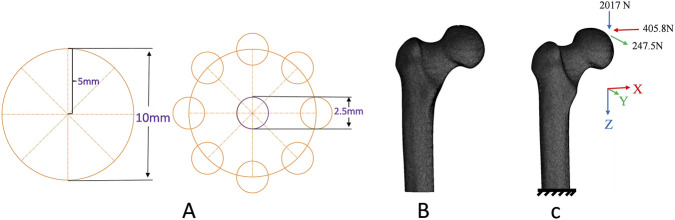
**(A)** Model design of multiple small diameter fan-shaped and parallel core decompression: 10 mm-diameter circle unifying drilling number distribution, evenly divided into eight sections from center, with 2.5 mm-diameter holes at center and around circle for consistent spacing; **(B)** Meshed model of proximal femur; **(C)** Boundary conditions.

**FIGURE 3:**
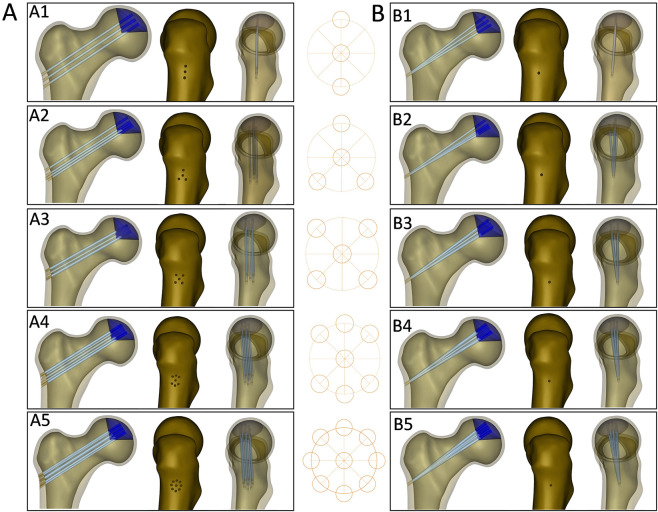
**(A)** Models of multiple small diameter parallel core decompression, A1: three-hole; A2: four-hole; A3: five-hole; A4: seven-hole; A5: nine-hole. **(B)** Models of multiple small diameter fan-shaped core decompression, B1: three-hole; B2: four-hole; B3: five-hole; B4: seven-hole; B5: nine-hole.

Ten finite element models (FEMs) were constructed using the SolidWorks 2022 software based on fan-shaped and parallel core decompression methods. The small-diameter core decompression was set at 2.5 mm. In order to ensure the consistency of the FEMs, the distribution of the drilling number was unified by using circles. The circle was set with a diameter of 10 mm and evenly divided into eight sections through its center. 2.5 mm diameter holes were evenly distributed at the center and around the circle, ensuring that the distances between the holes are consistent (Figure 2A). Models of parallel core decompression: Different numbers of small-diameter parallel core decompression (three-hole, four-hole, five-hole, seven-hole, and nine-hole) with the central hole kept unchanged (Figure 3A). Models of fan-shaped core decompression: the small-diameter core decompression was distributed in a fan-shape (all core decompression converged at a single hole on the greater trochanter’s lateral wall) and constructed different numbers of small-diameter core decompression with the central holes kept unchanged (three-hole, four-hole, five-hole, seven-hole, and nine-hole) (Figure 3B). In addition, control groups (no drilling and single 8 mm large-diameter drilling) were set up.

### 2.3 Finite element biomechanical analysis

The solid models were discretized into ten-node tetrahedral elements (solid187) using the ANSYS Workbench 2022 software (ANSYS, American). The model was validated by comparison with published literature and results demonstrate that the models are reliable and suitable for further finite element analysis (Yuan et al., 2022b). A sensitivity analysis was conducted to determine the optimal element size for the model (Jones and Wilcox, 2008). The model of three-hole parallel core decompression was used to construct FEMs comprising five different element sizes (0.6, 0.8, 1.0, 1.2, and 1.5 mm). Then the maximum equivalent stress in the greater trochanter’s lateral wall was compared. The percentage change in the normal walking load between the 0.8 mm model and the 1 mm model was <0.5%. Even the finest mesh-0.6 mm did not result in a significant percentage difference. The results remained stable and convergent with refined meshes, indicating that they are reliable and can be used for credible finite element analysis. Therefore, the 1 mm mesh was used for the FEMs. There were approximately 320,0000 nodes (from 3,237,050 to 3,259,150) and 2,300,000 elements (from 2,288,043 to 2,311,092) in each model. All materials were assumed to be homogeneous, isotropic, and with linear elastic behavior (Brown et al., 1981; Wirtz et al., 2000) (Table 1). The type of mechanical contact was bonded. The elliptical area at the junction of the femoral head and the acetabulum was set the weight-bearing area. The normal walking was assigned to the femoral head according to a previous study and the distal femur was fully restrained to the movement (Bergmann et al., 2001) (Figures 2B,C).

**TABLE 1:** Material properties of all modes in this study.

Materials	Young’s modulus (MPa)	Poisson’s ratio
Cortical bone	15,100	0.3
Cancellous bone	445	0.22
Early ONFH	332.9	0.3
Articular cartilage	150	0.2

ONFH, osteonecrosis of the femoral head.

### 2.4 Evaluation indices

Evaluation indices included the maximum equivalent stress, average stress, and local strain of the proximal femur (the entire structure, the greater trochanter’s lateral wall, and the bone tunnel) to evaluate the biomechanical stability.

## 3 Results

### 3.1 Stress distribution and the maximum stress of the entire structure and the greater trochanter’s lateral wall

**FIGURE 4:**
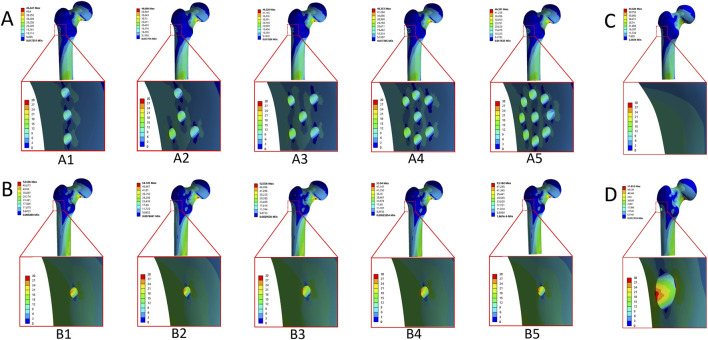
The von Mises stress distribution of the entire structure and the greater trochanter’s lateral wall. **(A)** Parallel drilling, A1: Three-hole; A2: Four-hole; A3: Five-hole; A4: Seven-hole; A5: Nine-hole. **(B)** Fan-shaped drilling, B1: Three-hole; B2: Four-hole; B3: Five-hole; B4: Seven-hole; B5: Nine-hole. **(C)** No drilling. **(D)** Single large-diameter drilling.

**FIGURE 5:**
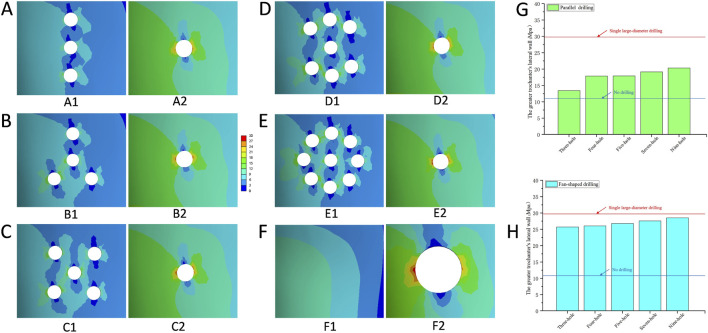
The von Mises stress distribution and the comparison of the maximum equivalent stress in the greater trochanter’s lateral wall. **(A)** Three-hole, A1: Parallel drilling; A2: Fan-shaped drilling. **(B)** Four-hole, B1: Parallel drilling; B2: Fan-shaped drilling. **(C)** Five-hole, C1: Parallel drilling; C2: Fan-shaped drilling. **(D)** Seven-hole, D1: Parallel drilling; D2: Fan-shaped drilling. **(E)** Nine-hole, E1: Parallel drilling; E2: Fan-shaped drilling. **(F)** No drilling and Single large-diameter drilling. **(G)** The comparison of the maximum equivalent stress (Parallel drilling). **(H)** The comparison of the maximum equivalent stress (Fan-shaped drilling) (Red line: single large-diameter drilling; blue line: no drilling).

Maximum equivalent stress values for the entire proximal femoral structure and the greater trochanter’s lateral wall (GLW) under different drilling configurations are presented in Table 2. In the no-drilling model, the maximum equivalent stress of the entire structure was 44.42 MPa, with a corresponding GLW stress of 10.80 MPa; stress contour plots showed a relatively uniform stress distribution across the GLW. Single large-diameter drilling (SLD) increased these values to 51.89 MPa and 29.56 MPa, respectively, with contour plots revealing prominent stress concentration at the periphery of the single large tunnel. In the parallel drilling group, maximum equivalent stress values for the entire structure and GLW ranged from 45.45 MPa to 13.45 MPa (three-hole) to 46.39 MPa and 20.36 MPa (nine-hole), with stress distributed across multiple small tunnels and increasing gradually with hole number. In the fan-shaped drilling group, these values ranged from 52.51 MPa to 25.72 MPa (three-hole) to 53.16 MPa and 28.51 MPa (nine-hole), with more pronounced stress concentration and higher overall stress magnitudes compared to parallel drilling at an equivalent number of holes. Bar charts confirmed that both drilling configurations resulted in higher GLW stress relative to the no-drilling model, with fan-shaped drilling consistently producing higher stress values than parallel drilling at each hole count, and the nine-hole fan-shaped configuration yielding the highest stress values among all tested groups (Figures 4, 5).

**TABLE 2:** The maximum equivalent stress at each part of the proximal femur (MPa).

Models	Entire structure	GLW	Cortical bone tunnel	Cancellous bone tunnel	BTOA
No drilling	44.42	10.80	​	​	​
SLD	51.89	29.56	29.56	2.67	2.51
Parallel drilling					
Three-hole	45.45	13.45	13.45	2.03	1.39
Four-hole	46.09	17.86	17.86	2.11	1.55
Five-hole	46.33	17.91	17.91	2.24	1.59
Seven-hole	46.35	19.18	19.18	2.58	1.70
Nine-hole	46.39	20.36	20.36	2.59	1.83
Fan-shaped drilling					
Three-hole	52.51	25.72	25.72	2.46	1.69
Four-hole	52.73	26.08	26.08	7.62	1.89
Five-hole	52.84	26.77	26.77	7.94	1.98
Seven-hole	53.04	27.58	27.58	12.20	2.25
Nine-hole	53.16	28.51	28.51	17.76	2.37

SLD, single large-diameter drilling; GLW, Greater trochanter’s lateral wall; BTOA, bone tunnel in the osteonecrosis area.

### 3.2 Stress distribution and the maximum stress of the bone tunnel

**FIGURE 6:**
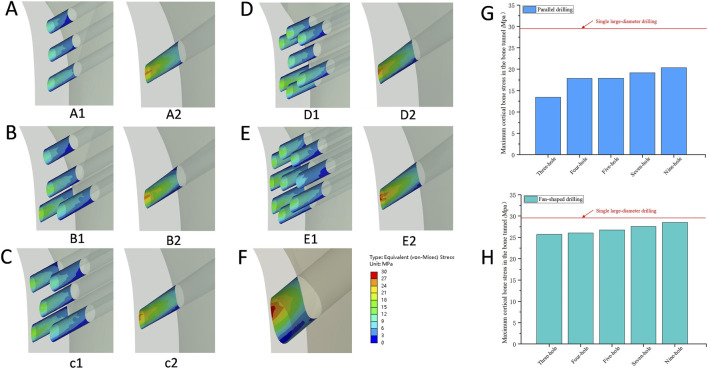
The von Mises stress distribution and the comparison of the maximum equivalent stress in the cortical bone tunnel. **(A)** Three-hole, A1: Parallel drilling; A2: Fan-shaped drilling. **(B)** Four-hole, B1: Parallel drilling; B2: Fan-shaped drilling. **(C)** Five-hole, C1: Parallel drilling; C2: Fan-shaped drilling. **(D)** Seven-hole, D1: Parallel drilling; D2: Fan-shaped drilling. **(E)** Nine-hole, E1: Parallel drilling; E2: Fan-shaped drilling. **(F)** Single large-diameter drilling. **(G)** The comparison of the maximum equivalent stress (Parallel drilling). **(H)** The comparison of the maximum equivalent stress (Fan-shaped drilling) (Red line: single large-diameter drilling).

**FIGURE 7:**
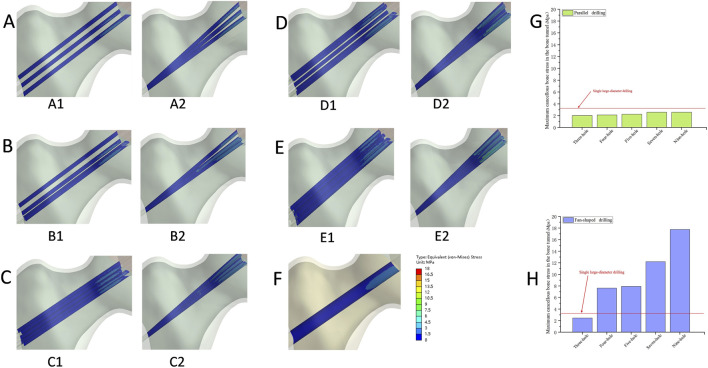
The von Mises stress distribution and the comparison of the maximum equivalent stress in the cancellous bone tunnel. **(A)** Three-hole, A1: Parallel drilling; A2: Fan-shaped drilling. **(B)** Four-hole, B1: Parallel drilling; B2: Fan-shaped drilling. **(C)** Five-hole, C1: Parallel drilling; C2: Fan-shaped drilling. **(D)**Seven-hole, D1: Parallel drilling; D2: Fan-shaped drilling. **(E)** Nine-hole, E1: Parallel drilling; E2: Fan-shaped drilling. **(F)** Single large-diameter drilling. **(G)** The comparison of the maximum equivalent stress (Parallel drilling). **(H)** The comparison of the maximum equivalent stress (Fan-shaped drilling) (Red line: single large-diameter drilling).

**FIGURE 8:**
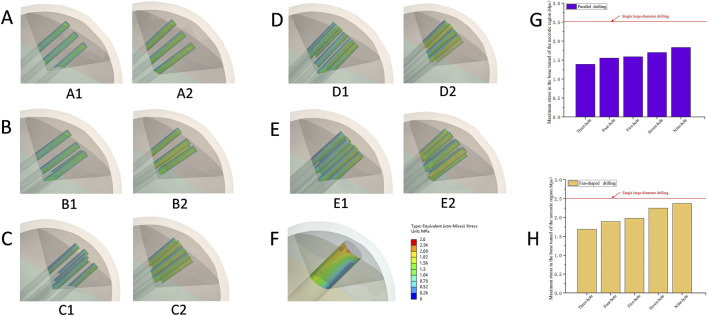
The von Mises stress distribution and the comparison of the maximum equivalent stress in the bone tunnel of the necrotic area. **(A)** Three-hole, A1: Parallel drilling; A2: Fan-shaped drilling. **(B)** Four-hole, B1: Parallel drilling; B2: Fan-shaped drilling. **(C)** Five-hole, C1: Parallel drilling; C2: Fan-shaped drilling. **(D)** Seven-hole, D1: Parallel drilling; D2: Fan-shaped drilling. **(E)** Nine-hole, E1: Parallel drilling; E2: Fan-shaped drilling. **(F)** Single large-diameter drilling. **(G)** The comparison of the maximum equivalent stress (Parallel drilling). **(H)** The comparison of the maximum equivalent stress (Fan-shaped drilling) (Red line: single large-diameter drilling).

As shown in Table 2, Representative von Mises stress distributions in the cortical bone tunnel (Figure 6) revealed that the single large-diameter drilling (SLD) model exhibited a maximum equivalent stress of 29.56 MPa, with high-stress regions concentrated at the tunnel’s proximal entry; in parallel drilling models (Figures 6A–C), stress increased incrementally with the number of holes (13.45–20.36 MPa), while fan-shaped drilling models (Figures 6D,E) displayed consistently higher cortical tunnel stress (25.72–28.51 MPa) with distributions similar to SLD (Figures 6F-H). In the cancellous bone tunnel (Figure 7), parallel drilling models (Figures 7A–C) maintained uniformly low stress (2.03–2.59 MPa), whereas fan-shaped drilling models (Figures 7D,E) showed a marked increase in stress with the number of holes (2.46–17.76 MPa), with high-stress regions concentrated at the tunnel’s distal end, and the SLD model exhibited a comparable stress of 2.67 MPa (Figures 7F-H). For the bone tunnel in the osteonecrosis area (BTOA, Figure 8), both parallel and fanshaped drilling models (Figures 8A–E) maintained relatively low stress (1.39–1.83 MPa and 1.69–2.37 MPa, respectively) with no significant stress concentration, while the SLD model (Figure 8F) showed a BTOA stress of 2.51 MPa, comparable to the maximum values observed in multi-hole drilling models (Figures 8G,H) (Table 2).

### 3.3 Average stress and local strain of the bone tunnel

**FIGURE 9:**
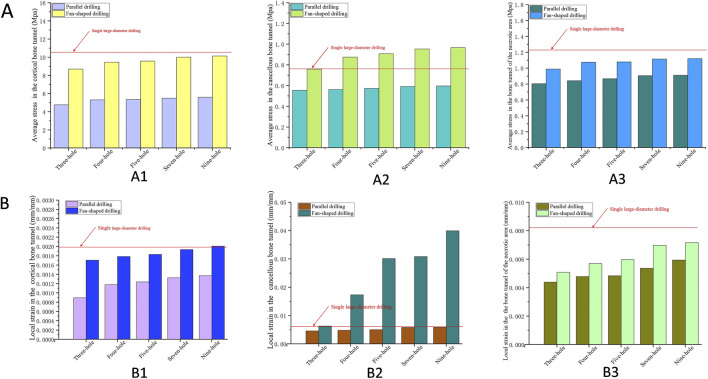
The average stress and local strain across key regions. **(A)** The average stress across key regions, (A1) The cortical bone tunnel; (A2) The cancellous bone tunnel; (A3) The bone tunnel of the necrotic area. **(B)** Local strain across key regions, (B1) The cortical bone tunnel; (B2) The cancellous bone tunnel; (B3) The bone tunnel of the necrotic area.

As shown in Table 3, average stress in the cortical bone tunnel, cancellous bone tunnel, and bone tunnel in the osteonecrosis area (BTOA) varied with drilling configuration. Single large-diameter drilling yielded the highest cortical bone tunnel stress (10.28 MPa). For parallel drilling, stress in all three regions increased gradually with more holes (three to nine-holes: cortical: 4.76–5.60 MPa; cancellous: 0.556–0.597 MPa; BTOA: 0.805–0.911 MPa). Fan-shaped drilling produced higher stress across all regions than parallel drilling at the same hole count (three to nine-holes: cortical: 8.69–10.14 MPa; cancellous: 0.760–0.967 MPa; BTOA: 0.991–1.121 MPa), with cortical bone tunnel stress approaching that of single large-diameter drilling (Figure 9A). As shown in Table 4, local strain varied with drilling configuration. For parallel drilling, strain increased with more holes. Fan-shaped drilling produced notably higher strain, especially in the cancellous bone tunnel, compared to parallel drilling, with cortical bone tunnel strain approaching that of single large-diameter drilling (Figure 9B).

**TABLE 3:** The average stress of the bone tunnel (MPa).

Models	Cortical bone tunnel	Cancellous bone tunnel	BTOA
Single large-diameter drilling	10.28	0.772	1.23
Parallel drilling			
Three-hole	4.76	0.556	0.805
Four-hole	5.30	0.561	0.843
Five-hole	5.34	0.574	0.868
Seven-hole	5.49	0.592	0.906
Nine-hole	5.60	0.597	0.911
Fan-shaped drilling			
Three-hole	8.69	0.760	0.991
Four-hole	9.46	0.876	1.074
Five-hole	9.58	0.908	1.079
Seven-hole	10.00	0.954	1.115
Nine-hole	10.14	0.967	1.121

BTOA, bone tunnel in the osteonecrosis area.

**TABLE 4:** The local strain of the bone tunnel (mm/mm).

Models	Cortical bone tunnel	Cancellous bone tunnel	BTOA
Single large-diameter drilling	0.00197	0.006006	0.00810
Parallel drilling			
Three-hole	0.00090	0.00458	0.00440
Four-hole	0.00118	0.00479	0.00479
Five-hole	0.00124	0.00505	0.00484
Seven-hole	0.00133	0.00583	0.00538
Nine-hole	0.00137	0.00587	0.00594
Fan-shaped drilling			
Three-hole	0.00171	0.00629	0.00508
Four-hole	0.00179	0.01733	0.00570
Five-hole	0.00183	0.03015	0.00598
Seven-hole	0.00194	0.03083	0.00698
Nine-hole	0.00201	0.03995	0.00717

BTOA, bone tunnel in the osteonecrosis area.

## 4 Discussion

In this study, the finite element method was used to compare the effects of the different distribution parameters of the multiple small-diameter fan-shaped and parallel core decompression for early ONFH on the biomechanical stability after surgery. The results showed that the parallel core decompression could disperse the stress transmitted downward from the femoral head through the proximal femur, avoiding the stress concentration of the greater trochanter’s lateral wall and the bone tunnel and increasing the local mechanical stability, in comparison to the fan-shaped core decompression.

Human bone is an elastic material, plastic deformation occurs when it is subjected to external stress, which can change the distribution of stress (Morgan et al., 2018). In this study, the core decompression procedure damaged the cortical bone on the lateral wall of the greater trochanter, resulting in changes to its original mechanical structure and alterations in stress distribution. Even stress concentration phenomena occurred at the tunnel opening. Stress concentration refers to the phenomenon of local increase of stress in the object, which generally appears in the place where the object shape changes rapidly and can cause fatigue cracks in the object (Burr, 2019). In this study, the convergence of the multiple small diameter fan-shaped core decompression tended to form irregular bone tunnel shapes. Moreover, as was seen from Table 2, when comparing the stress conditions at different locations, the stress values of the multiple small diameter fan-shaped core decompression were relatively larger, and the stress concentration phenomenon was more obvious, which was similar to the aforementioned research. The recurrence of fracture after internal fixation is closely related to stress concentration, which leads to a greater stress value at the local fracture site than at other locations, closer to or more than the yield strength of the bone, increasing the risk of fracture (Samsami et al., 2019). Under physiological conditions, the load transmitted from the femoral head to the neck does not follow a linear path due to the femoral neck-shaft angle and anterior torsion angle. This generates tensile, compressive, and shear stresses at the femoral neck, primarily concentrated in the lower part of the femoral head-neck joint (Miyachi et al., 2019). This is consistent with our research results, as the model did not alter the stress distribution pattern in the area below the femoral neck.

In this study, the bone tunnels of the multiple small diameter fan-shaped core decompression extended convergently from the femoral head to the lateral wall of the greater trochanter. The final formed bone tunnels present an irregular geometric shape due to the intersection of each path in space. As be seen from the data in Figure 3, with the increase in the number of fan-shaped bone holes, the degree of irregularity of the bone tunnel morphology in the convergence area was significantly intensified. Irregular geometric structures are extremely prone to causing stress concentration during the stress conduction process based on the stress-strain theory in material mechanics (De Stefano et al., 2023). The data in Table 2 also showed that the stress value of the fan-shaped core decompression group had a significant positive correlation with the number of bone holes, that was, with the increase in the number of fan-shaped bone holes, the stress value borne by the bone tunnel showed a gradual increasing trend. The bone tunnels of the parallel core decompression group were spatially parallel, and there was no intersection between each bone tunnel, so the bone tunnel structure formed in the necrotic area, cancellous bone and cortical bone of the lateral wall of the trochanter was uniform and regular. This regular geometric shape effectively reduced the risk of stress concentration, and its stress value was smaller than that of the multiple small diameter fan-shaped core decompression group. The results are in agreement with the finite element theory (Pérez-Pevida et al., 2021), verifying the advantage of parallel core decompression in controlling stress distribution. The conclusions of this study are applicable to the achievable range of no more than nine holes under actual clinical or experimental conditions. Within this range, the biomechanical advantages of parallel core decompression with regard to stress distribution and mechanical stability remain valid and reliable. If exceeding this range, the biomechanical benefits observed in the present study should not be directly extrapolated to an excessively high number of holes that cannot be achieved in practice, since excessive drilling may lead to unnecessary bone loss, decreased structural strength, and potential injuries.

The present study had some limitations. First, in this study, the material properties were assumed to be isotropic, linearly elastic, and homogeneous. In reality, ONFH lesions vary greatly in morphology, location, size, and their relationship to the weight-bearing zone. This model may not represent all clinical scenarios. Secondly, the study did not consider the biological interface between necrotic and viable bone. The sclerotic rim is typically mechanically stronger and may significantly alter stress transmission paths, which could bias the model’s stress distribution compared with reality. Thirdly, only a single static peak gait load was applied in the loading scheme. The hip joint experiences complex, multi-directional dynamic loads in daily activities. Using a unidirectional load cannot fully assess the risk of fatigue failure in the drilled femur, particularly within the bone tunnels, under complex stress states. Finally, the study presents differences and percentage changes in stress values between groups but does not employ any statistical methods to examine whether these differences are statistically significant. In the future, we expect to conduct more realistic biomechanical experiments and clinical trials to verify our results.

## 5 Conclusion

From a biomechanical perspective, multiple small-diameter parallel core decompression provides better biomechanical stability and reduces stress concentration in the proximal femur compared to fan-shaped core decompression, making it a more favorable option for early osteonecrosis of the femoral head.

## Data Availability

The raw data supporting the conclusions of this article will be made available by the authors, without undue reservation.
